# Quantification of global myocardial oxygenation in humans: initial experience

**DOI:** 10.1186/1532-429X-12-34

**Published:** 2010-06-02

**Authors:** Kyle S McCommis, Robert O'Connor, Donna Lesniak, Matt Lyons, Pamela K Woodard, Robert J Gropler, Jie Zheng

**Affiliations:** 1 Mallinckrodt Institute of Radiology, Washington University School of Medicine, 510 S. Kingshighway Blvd, St. Louis, MO 63110, USA

## Abstract

**Purpose:**

To assess the feasibility of our newly developed cardiovascular magnetic resonance (CMR) methods to quantify global and/or regional myocardial oxygen consumption rate (MVO_2_) at rest and during pharmacologically-induced vasodilation in normal volunteers.

**Methods:**

A breath-hold T_2 _quantification method is developed to calculate oxygen extraction fraction (OEF) and MVO_2 _rate at rest and/or during hyperemia, using a two-compartment model. A previously reported T_2 _quantification method using turbo-spin-echo sequence was also applied for comparison. CMR scans were performed in 6 normal volunteers. Each imaging session consisted of imaging at rest and during adenosine-induced vasodilation. The new T_2 _quantification method was applied to calculate T_2 _in the coronary sinus (CS), as well as in myocardial tissue. Resting CS OEF, representing resting global myocardial OEF, and myocardial OEF during adenosine vasodilation were then calculated by the model. Myocardial blood flow (MBF) was also obtained to calculate MVO_2_, by using a first-pass perfusion imaging approach.

**Results:**

The T_2 _quantification method yielded a hyperemic OEF of 0.37 ± 0.05 and a hyperemic MVO_2 _of 9.2 ± 2.4 μmol/g/min. The corresponding resting values were 0.73 ± 0.05 and 5.2 ± 1.7 μmol/g/min respectively, which agreed well with published literature values. The MVO_2 _rose proportionally with rate-pressure product from the rest condition. The T_2 _sensitivity is approximately 95% higher with the new T_2 _method than turbo-spin-echo method.

**Conclusion:**

The CMR oxygenation method demonstrates the potential for non-invasive estimation of myocardial oxygenation, and should be explored in patients with altered myocardial oxygenation.

## Introduction

As an aerobic organ, the heart has to consume large amounts of O_2 _for its primary contractile function. Oxygen supply and demand must match to maintain normal myocardial contractility. The myocardial oxygen extraction fraction (OEF = ([O_2_]_artery _- [O_2_]_venous_)/[O_2_]_artery_) reflects this balance in the heart. Non-invasive assessments of this parameter are of paramount interest because they may provide convenient and cost-effective tools to study the pathophysiology and metabolic consequences of myocardial ischemia. While progress has been made in the qualitative assessment of myocardial oxygenation with myocardial T_2 _or T_2_* contrast [1-3], limited effort has been devoted to quantification of myocardial OEF by cardiovascular magnetic resonance (CMR), particularly on a regional basis. A quantification model was recently reported to measure myocardial OEF during pharmacologically induced hyperemia in vivo using myocardial BOLD effects [4,5]. The model was validated in animal studies, using a segmented black-blood Turbo-Spin-Echo (TSE) sequence with T_2 _contrast. However, this CMR T_2 _based oximetry technique has yet been assessed in human subjects. Furthermore, irregular ECG triggering due to tachycardia or arrhythmias, and unsaturated blood flow artifacts, which often occur in cardiac patients, may deteriorate the T_2_-weighted image quality using the TSE sequence.

The overall goal of this study was to translate our validated CMR oximetry technique to human imaging. In particular, we investigated a modified T_2 _preparation technique (T_2_prep) that can be performed in human subjects with reduced susceptibility to the adverse ECG-triggering effects aforementioned and improved sensitivity to myocardial oxygenation alternations. Similar methods were reported recently to qualitatively assess myocardial oxygenation [6,7]. Like the quantification model, it utilizes T_2_-weighted contrasts. However, by selecting proper imaging parameters, the imaging procedure can be simplified compared to the TSE methods. Moreover, the OEF at the coronary sinus can be quantified using the T_2_prep sequence as an index of global myocardial OEF at rest, which was an assumed value in the previous animal studies [4,5]. Lastly, unlike the intravascular contrast agent used in the previous animal studies, we will utilize an FDA-approved extracellular agent, Multihance, for myocardial perfusion quantification which is used for the measurement of myocardial oxygen consumption rate (MVO_2_). The CMR T_2_prep oximetry method was demonstrated in human volunteers, at rest and during adenosine-induced vasodilation. The findings from the T_2_prep method were compared with the previously reported TSE method for the quantification of myocardial OEF during adenosine vasodilation.

## Theory and Methods

### Theory

The detail of the model was reported previously [4]. Briefly speaking, in T_2_-weighted TSE or spin-echo (SE) images with an interecho spacing τ (the time difference between two consecutive 180° pulses in TSE or between the 90° pulse and subsequent echoes in SE), the signal in a myocardial tissue voxel can be approximated in a biexponential form from a two-compartment model as follows:(1)

where *S*_voxel _is the mean signal intensity of the voxel at echo time TE,; *S*_*0 *_is a variable related to the proton density of the voxel, receiver gain, and T_1 _of the tissue. T_2b _and T_2t _are the T_2 _values of blood and tissue, respectively. Using the van Zijl intravascular component model [8], intravascular T_2 _can be derived:(2)

where α_1_, α_2_, and α_3 _are the functions of magnetic susceptibilities, interecho spacing τ, oxygenation-dependent T_2 _of erythrocytes and plasma, arterial oxygen saturation, and hematocrit. The three constants can be derived with experimental data obtained at 1.5 T [9], using subject-specific hematocrit values. For coronary sinus imaging, only the first term in Eq. [1] was used. The extravascular T_2t _can be approximated using a diffusion model [10,11]:(3)

where R_20t _is the intrinsic myocardial tissue transverse relaxation rate, and R_21t _is a function of the diffusion constant (D), susceptibility difference between the blood vessel and the surrounding tissue, geometry of the heart relative to the B_0 _static field, and the size of capillary and venous vessels. Therefore, both R_20t _and R_21t _are subject-specific parameters and need to be determined during each imaging session. Increasing τ_180 _would increase the sensitivity of this method to the changes of myocardial oxygenation. With knowledge of R_20t_, R_21t_, apparent myocardial T_2_, and MBV, hyperemic myocardial OEF can be calculated through Eqs. (1-3).

### T_2_prep Imaging Method for Myocardial OEF Estimation

**Figure 1:**
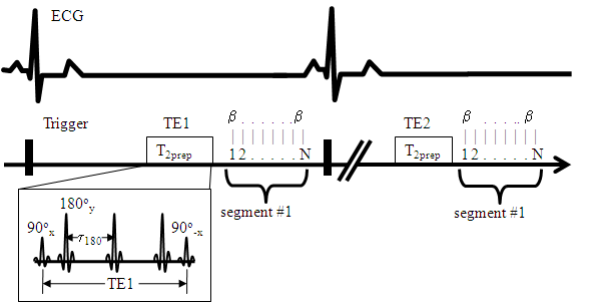
**Diagram of T2prep sequence for the measurement of myocardial T_2_-weighted images**. The sequence begins with a T2prep module followed by a N-line of gradient-echo data acquisition using a flip angle of β. All pulses in T_2_prep module are composite pulses. The acquisition continues until the full k-space lines are collected. The second TE2 images are then acquired in the same manner, followed by the TE3, TE4, etc.

The T_2_prep sequence was implemented in a 1.5 T Siemens Sonata system (Siemens Medical Solutions, Malvern, PA) (Figure 1). The sequence consists of a T_2_prep module and gradient-echo acquisition module. The T_2_prep module maintains the flexibility of changing the number of 180° pulses between two 90° tip-down and tip-up pulses. No gradients were played during the T_2 _preparation period, except one large spoil gradient at the end of T_2_prep module. Both 90° and 180° pulses were composite pulses. The 90°_x _consisted of a (270°_x_)(360°_-x_) composite pulse, whereas the 180°_x _was consisted of a (90°_x_)(180°_y_) (90°_x_) composite pulse. The last tip-up 90° pulse was (360°_x_270°_-x_). If the TE is fixed, more 180° pulses will lead to less interecho spacing τ. In this study, because longer τ may increase the sensitivity of the sequence to the changes in myocardial oxygenation due to the extravascular diffusion weighting factor [5], it is desirable to have 180° pulses as less as possible in the T_2_prep module. A previous study [12] showed that a 4-180° scheme can minimize the B_1 _and B_0 _inhomogeneities. We observed (data not shown) that a T_2_prep module with three 180° pulses is sufficient to minimize inhomogeneity artifacts. The gradient-echo acquisition allows for bright-blood imaging, and thus reduces the flow artifacts. In addition, gradient-echo acquisition is less sensitive to cardiac motion compared to the TSE sequence.

The T_2_prep sequence was used to obtain T_2 _maps with different TE values. In the previous OEF model using the TSE acquisition, at least two different τ_180 _values were needed to calculate model parameters R_20t _and R_21t_, leading to two separate TSE acquisitions at rest. Using the T_2_prep method, only one acquisition is necessary since different TE or τ_180 _values are used for the T_2 _calculation. Therefore, we developed a multi-variable regression method for the data set {S_voxel_, TE, α_1_, α_2_, α_3_} at rest for the determination of R_20t _and R_21t_. Then using the data set during hyperemia, a similar multi-variable regression can be repeated again to calculate the hyperemic OEF. Table 1 shows the TE and the model parameters we used to fit Eq. (1-3) in this method. The way to derive these parameters was reported previously [5], which were based on the theoretical model by another group [8]. It is noted that these parameters in Table 1 are different between myocardium and CS due to the difference in hematocrit value [8]. The hematocrit was assumed to be one half of that in coronary sinus (40%), i.e. 20% [13]. Increasing τ_180 _will increase α_1_, α_2_, α_3_, and thereby increase the sensitivity of T_2 _to the change in OEF (Eq. 2 and 3). MBV at rest and during hyperemia were calculated independently by using first-pass perfusion imaging.

**Table 1:** Fitting Parameters for the Calculation of Coronary Sinus and Myocardial Hyperemic OEF

TE (ms)	Coronary Sinus			Myocardium		
	
	**α**_**1**_	**α**_**2**_	**α**_**3**_	**α**_**1**_	**α**_**2**_	**α**_**3**_
24	2.42	4.25	3.94	2.52	2.69	2.83
36	4.78	5.27	4.05	3.76	3.22	2.89
48	7.25	6.33	4.17	4.57	3.57	2.93
60	9.55	7.32	4.28	5.09	3.80	2.95
72	11.54	8.18	4.37	5.45	3.95	2.97

### OEF of Coronary Sinus

We estimated the resting global OEF using a similar method reported by Foltz et al [14]. Briefly, coronary sinus T_2 _was measured using the same T_2_prep imaging sequence. The resting OEF was determined by a blood T_2_-OEF model, expressed in Eq. [1-2] with the parameters defined in Table 1. Since 90% of left interventricle blood is drained to the coronary sinus, such measured resting OEF should represent the global OEF of the left ventricle.

### T_2 _Phantom Study

The T_2 _accuracy measured by the T_2_prep sequence was first studied in a phantom study. Four polyethylene tubes with a diameter of 3 cm were filled by a combination of agarose, Gd-DTPA, and NaCl, and distilled water. Such compositions created phantoms with T_1_, T_2_, and dielectric property similar to human myocardial tissues [15]. The concentrations of these components were slightly varied to yield T_2 _from 30 to 50 msec in 4 phantoms. Table 2 lists the T_1 _and T_2 _values of these phantoms. The T_2_prep sequence using the same imaging parameters as in volunteer study (see below) was applied to scan these phantoms. Standard spin echo sequence with 7 different TEs from 24 to 96 msec was used to provide reference T_2 _values.

**Table 2:** T_2 _phantoms and T_2 _measurement results with T_2_prep and SE sequences

Sequences	Phantom 1 (1043.5)	Phantom 2 (1122.4)	Phantom 3 (754.3)	Phantom 4 (859.5)
SE	32.3	43.9	46.5	51.4
T2prep	32.3	43.5	46	51.7
Errors (%)	0	-0.91	-1.0	0.6

T1 values are shown in parenthesis.

### Volunteer Study

**Figure 2:**
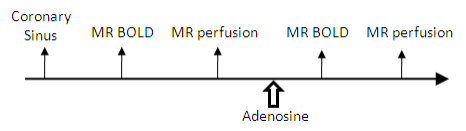
**Time sequence of human study protocol**.

Six healthy volunteers (average age = 32 ± 5 years old, 4 male) were recruited for this study. Informed consent was obtained from each volunteer prior to imaging session. Figure 2 shows the time sequence of the measurements. Coronary sinus imaging was performed first at rest, followed by T_2_-weighted imaging using both T_2_prep and TSE sequences. The location of the image plane was approximately 2-3 cm from the sinus ostium [14] to ensure the blood in the CS was dominated by the venous blood. We selected Multihance as the contrast agent for the first-pass perfusion imaging, partially because of its lowest risk profile to nephrogenic systemic fibrosis (NSF) [16]. Bolus injections of Multihance (0.02 mmol/kg) were performed two times using a MR compatible injector (Spectris, Medrad, Indianola, PA), once at rest and once 4-6 min after the start of infusion of adenosine for coronary artery vasodilation. The time interval between two contrast injections was 15 ± 2 min. This allowed for the washout of the first contrast agent to minimize the interference of two contrast injections.

Adenosine was infused intravenously for 6 minutes at a constant rate of 0.14 mg/kg/min by using a CMR compatible infusion system (Continuum, Medrad, Indianola, PA). Two minutes after the start of adenosine infusion, CMR started with T_2_-weighted imaging, followed by the first-pass perfusion imaging as the last scan. Heart rate (HR) and blood pressure (BP) were measured continuously during the entire image session with an CMR-compatible vital-signs monitoring system (Millennia; Invivo Research, Orlando, FL). The hard record was obtained at rest and every 1 min after the start of the adenosine infusion until the end of the infusion.

The T_2_-weighted imaging consisted of two imaging methods: T_2_prep and TSE. The image parameters for T_2_prep were as follows: TE in the T_2_prep module = 24, 36, 48, 60, 72 ms, gradient-echo readout TR/TE = 3.6/2.0 ms, segmentation number = 27-35 (depending on the RR interval), bandwidth = 400 Hz/pixel, slice thickness = 8 mm, data matrix = 145 × 256, FOV = 255 × 340 mm^2^, and flip-angle = 15°. The scan time was approximately 20-25 sec. For CS imaging, the spatial resolution was slightly higher, i.e., data matrix = 162 × 256, resulting in a resolution of 1.57 mm × 1.32 mm. For TSE imaging at rest, two scans were performed using two different interecho spacing τ values, i.e., 8 and 12 ms, to calculate parameters R_20t _and R_21t _in Eq. [3]. Other imaging parameters included: TE = 24, 48, and 72 ms, segmentation number = 5, bandwidth = 390 Hz/pixel, slice thickness = 8 mm, data matrix = 145 × 256, FOV = 255 × 340 mm^2^, scan time = 22-25 sec. Respiratory motion was controlled by breath-hold at end-expiration.

The first-pass perfusion sequence was a 2 D multi-slice saturation-recovery (SR) prepared Turbo FLASH sequence with a total of 70-80 images acquired in each slice. The T_1_-weighting was provided by a volume-selective SR pulse. Each dynamic image was obtained in mid-diastole by ECG triggering. The center slice was located approximately at the mid-cavity level of the left ventricle across the papillary muscles in a short-axis view. Image parameters were as follows: TR/TE = 2.4/1.2 ms, slice thickness = 8 mm, data matrix = 84 × 256, FOV = 137 × 220 mm^2^, flip-angle = 18°, and SR time = 100 ms depending on the RR interval. Respiratory motion was controlled by breath-hold at the end expiration.

### Image Processing and Statistical Analysis

The central slice of the perfusion imaging corresponding to the T_2_prep slice was processed using homemade software developed in our laboratory, written in JAVA (Java V5.0, Sun Microsystems, Santa Clara, CA). A model-free deconvolution method, Consolidated-basis, was implemented to calculate MBF and MBV [17]. The approach used a linear combination of basis functions to represent impulse function. Few parameters [3] are required for the deconvolution process and thus noise-sensitivity is relatively low. Using the mean transit time (MTT) determined by the software, MBV is calculated as the product of MTT and MBF. The software provides the capability for denoising the image before mapping MBF or MBV [18]. On each MBF map, a large, global ring ROI was drawn to include approximately 75% transmural left ventricle myocardial wall to calculate the global MBF. Input curves for these calculations were obtained from a circular ROI manually drawn over the left ventricle blood pool.

To process coronary sinus images, a circle ROI was drawn in the center of the coronary sinus and adjusted manually for the compensation of motions between different T_2_-weighted images. These signal intensities were then fitted to Eq. (1-2) using the constants defined in Table 1, without using the second term in Eq. (1). Resting CS OEF could be obtained by the multi-variable regression method using DataFit (Oakdale Engineering, Oakdale, PA).

Myocardial T_2_prep images were analyzed by drawing the same ring ROI as in the perfusion measurements and T_2_-weighted signal intensities at rest and during stress were obtained at different TEs. Theoretically, R_20t _and R_21t _can be determined using Eqs. (1-3) by using multi-variable non-linear regression procedures. However, it was found that non-linear fitting was not stable due to few data points for 3 parameters to be calculated. We have thus developed following procedures to calculate these parameters and myocardial regional OEF during stress:

(a) Using a single-variable non-linear regression, apparent T_2apparent _values are calculated at rest and during stress;

(b) With OEF obtained at rest from the CS measurement, R_2b _can be calculated at each TE using Eq. [2]; and R_2t _can be then calculated using Eq. [1] with MBV obtained from the perfusion measurement at rest.

(c) In Eq. [3], R_2t _is linearly correlated with R_20t _and R_21t _with the single variable OEF^2^MBV^2^τ^2^, where τ = TE/3. By linear fitting of Eq. [3], R_20t _(the intercept) and R_21t _(slope) can be calculated.

(d) With the knowledge of R_20t_, R_21t_, and MBV during stress, the relationship of T_2apparent _and OEF during stress can be derived using Eq. (1). Based on T_2apparent _during stress from (a), myocardial OEF during stress was obtained. The procedures for calculating hyperemic myocardial OEF using the TSE method were reported previously [2]. MVO_2 _was calculated using the formula: MVO_2 _(μmol/g/min) = 1.39 (mL O_2_/(hemoglobin) g) × 0.14 (hemoglobin) g/mL blood × 44.658 (μmol/mL) × 0.95 (Oxygen saturation in arterial blood) × OEF × MBF (mL/g/min). It should be noted, in contrast to the first-pass perfusion images, neither OEF nor MVO_2 _were calculated from maps, but from ROIs drawn on the source images.

To compare the performance of T_2_prep and TSE methods, T_2 _sensitivity was defined as(4)

It indicates the percentage change in measured myocardial T_2 _per 100% increases in MBF. All data are expressed as mean ± SD. Paired student's T-tests were performed to test significant differences between the TSE and T_2_prep methods, as well as between resting and hyperemic parameters. *P *< 0.05 was considered statistically significant.

## Results

### Phantom Study

The T_2 _measurement results are shown in Table 2 and the T_2 _error by using T_2_prep sequence was less than 1% in comparison with reference T_2 _values.

### MBF, OEF, and MVO_2 _of Global Myocardium at Rest

The volunteer's hemodynamic data along the time course is presented in Table 3, along with the measurement results of OEF of the coronary sinus at rest. HR and rate pressure product (RPP) were both significantly elevated over the resting values during adenosine infusion (~70% increase in RPP). Systolic blood pressure showed significant elevation only at 1 and 5 min after the start of adenosine infusion. The increase in RPP with the vasodilation agent adenosine is not unusual as this was also reported by others [19,20]. Figure 2 shows examples of T_2_-weighted images of the coronary sinus of one volunteer using the T_2_prep sequence. The resting CS T_2 _was 62 ± 4 msec and OEF was 0.73 ± 0.05, which agrees nicely with one previously reported global OEF of healthy volunteers (0.71 ± 0.08) measured by PET imaging [21]. The mean resting global MBF was 0.89 ± 0.36 mL/g/min and MBV was 5.5 ± 1.8 mL/100 g tissue. The corresponding resting MVO_2 _is approximately 5.2 ± 1.7 μmol/g/min or 0.116 ± 0.038 mL/g/min which also agrees well with published data in normal humans [18,22].

**Table 3:** Hemodynamic Data of the Volunteers and Resting CS OEF

	Heart Rate	Systolic Pressure	Rate-Pressure Product	Resting OEF
Rest	72 ± 11	113 ± 6	8102 ± 1273	0.73 ± 0.03
Adenosine	110 ± 16†	120 ± 13	13878 ± 1201†	

Values are presented as mean ± SD

†*P *< 0.001 for the comparison between rest and adenosine

### *MBF, OEF, and MVO_2 _of Global Myocardium during Adenosine Stress*

**Figure 3:**
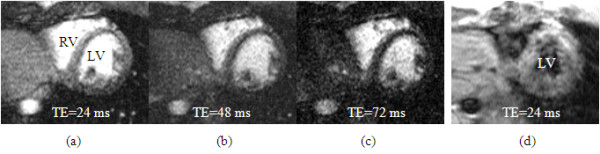
**Examples of bright blood T2-weighted myocardial images at three different TEs, obtained from a volunteer during the adenosine infusion**. In comparison, the TSE images collected is distorted by the fast heart rate and flow artifacts, which is not useful for further analysis.

One set of hyperemic BOLD images with the TSE method was distorted in one volunteer due to rapid changes in heart rate during adenosine infusion, but T_2_prep scans were completed in all volunteers. Figure 3 demonstrates three T_2_-weighted images using T_2_prep sequence and one distorted TSE images during the adenosine infusion. Table 4 shows the comparison of myocardial perfusion and oxygenation parameters from both the TSE and T_2_prep methods. Except for MVO_2 _obtained by the TSE method, significant differences were found between adenosine and resting measurements for all other parameters.

**Table 4:** Global Myocardial Perfusion and Oxygenation in Volunteers (Data is presented as mean (SD))

Method		T_2_	MBF (mL/g/min)	MBV (mL/g)	OEF	MVO_2_ (μmol/g/min)	T_2_sen*
T_2_prep	Rest	44 (6)	0.89 (0.36)	0.06 (0.02)	0.73 (0.05)	5.2 (1.7)	
	Aden	49 (5)†	2.97 (0.75)†	0.10 (0.02)†	0.37 (0.05)†	9.2 (2.4) ^ξ^	4.8(5.1)

TSE	Rest	55 (4)††			0.73 (0.05)	5.2 (1.7)	
	Aden	59 (4) †			0.41(0.14)†	10.4 (5.1)	3.2(2.9)

OEF at rest are values of coronary sinus in Table 2

MBF and MBV were calculated from the first-pass perfusion imaging data sets.

*T_2_sen or T_2 _sensitivity = percentage of T_2 _change/100% increase in MBF

Aden = Adenosine vasodilation

†*P *< 0.01 for comparison between rest and adenosine.

^ξ ^*P *< 0.05 for comparison between rest and adenosine.

††*P *< 0.05 for comparison between T_2_prep and TSE methods.

The mean hyperemic MBF was 2.97 ± 0.75 mL/g/min and MBV was 9.9 ± 2.4 mL/100 g. Adenosine induced 220 ± 152% increase in MBF and 65 ± 53% increase in MBV. The corresponding global myocardial T_2 _changes were 5.8 ± 2.0 % with the TSE method and 11.5 ± 8.1% with the T_2_prep method. Global myocardial T_2 _values are generally lower with T_2_prep method than TSE, due to the diffusion effects with larger echo spacing time in the T_2 _preparation pulses (8). The T_2 _sensitivity is approximately 95% higher with the T_2_prep method than TSE method, although no significant difference was observed due to limited subject number. The R_20t_, representing intrinsic myocardial tissue R_2_, was 24.5 ± 4.1 sec^-1^. The R_21t_, reflecting geometry of the heart and local gradient fields, was 10.6 ± 9.7 (×10^4^) sec^-3^. For the T_2_prep method, myocardial OEF was reduced 47 ± 21% during adenosine infusion and mean MVO_2 _increased approximately 88 ± 75%. This magnitude increase is consistent with the increase in the RPP value (71%). Overall, there were no significant differences in OEF and MVO_2 _measurements between the T_2_prep and TSE methods.

## Discussion

A modified quantitative CMR method was demonstrated to estimate global myocardial OEF and MVO_2 _in humans, at rest and during pharmacologically induced vasodilation. While resting OEF and MVO_2 _are only measurable on coronary sinus, regional myocardial OEF and MVO_2 _can be measured during hyperemia using myocardial BOLD effects. Adenosine induced 2-3 fold increases in MBF that exceeded the increase in MVO_2_. Consequently, myocardial OEF was reduced 47% in normal volunteers. The bright-blood T_2_prep method appears to be a better method than black-blood TSE technique, as indicated in much improved T_2 _sensitivity to the changes in myocardial blood flow (Table 4).

The models to estimate hyperemic myocardial OEF and MVO_2 _were established and validated in animal studies previously, using a black-blood TSE sequence [4,5]. However, in some cases, particularly in the presence of tachycardia or arrhythmias, black-blood preparation using the double inversion technique does not sufficiently suppress the blood signals in the left ventricle, resulting in flow artifacts on the myocardium. This often leads to decreased myocardial T_2 _and thus overestimation of myocardial OEF. The bright-blood T_2_prep technique, followed by robust gradient-echo acquisition, avoids these flow artifacts. Unlike the procedure to estimate hyperemic OEF using the TSE sequence [4,5], in which two resting scans are required to estimate model parameters R_20t _and R_21t_, the use of the T_2_prep sequence requires only one resting scan for this purpose. Up to five images with different echo times (TEs) can be acquired within one breath-hold rather than only 3 images using the TSE sequence, potentially increasing the precision of the OEF estimation. With the use of larger 180° echo time (τ), the sensitivity of the T_2_prep method to the changes of MBF seems to be improved. Finally, the simple adjustment of segmentation number for the gradient-echo acquisition allows for the change in breath-holding time, which is very beneficial for scanning patients in a clinical setting.

While the T_2 _preparation method to measure myocardial and coronary sinus T_2 _is not a new approach [13,23], the sequence developed allowed one T_2 _mapping with single breath-hold using up to 5 different TE. This approach is different from reported methods that used 2 to 3 TEs and/or scan for a relatively long time (~8 min). Another difference is the gradient-echo readout in the sequence, in comparison with reported readout sequences using spiral or TrueFISP. Apparently, gradient-echo has relatively lower SNR than the other two methods, but it offers the most robust signal acquisition against cardiac motion. Spiral readout was not available in our scanner. TrueFISP readout was not adopted due to following reasons: a) unlike the gradient echo sequence, data acquisition of TrueFISP readout starts at approximately 50 msec after the last 90° pulse in the T_2 _preparation module. This time delay reduced the efficiency of T_2 _weighting; b) steady-state TrueFISP signal is T_2_/T_1 _weighting. Because our model to calculate myocardial or CS OEF relies on T_2 _measurement, the impact of this T_2_/T_1 _weighting remains to be determined for the accuracy of OEF measurements. Nevertheless, TrueFISP type of readout is still an attractive option owing to its higher SNR. Further investigation of this sequence for the quantification of myocardial OEF is warranted.

Measurement of coronary venous oxygenation was previously reported by Foltz et al [13] and Yang et al [24] using a similar T_2_prep (MLEV module) with a spiral readout data acquisition. Four 180° pulses were used within the T_2_prep module to minimize B_0 _and B_1 _inhomogeneities in a previous study [12]. The coronary sinus oxygen content in %-O_2 _was calculated through an *in vitro *calibration procedure involving venipuncture and ex vivo CMR T_2 _determination in the blood samples. We have simplified this procedure using a previously established quantification model and directly calculated oxygen content of coronary sinus in OEF term by acquisitions of multiple T_2_-weighted signals and nonlinear fitting to the model equation. The resulted resting OEF appears to be consistent with other literature values [18]. It is noted that we used one or three 180° pulses in the T_2_prep module, aiming to maximize the diffusion sensitivity of the sequence to the changes in blood oxygenation. To effectively minimize B_0 _and B_1 _inhomogeneity, the combination of MLEV-4 pattern and composite pulses in the T_2_prep module may still be the optimal condition [12]. Our sequence can be prescribed to using 4 180° pulses in the T_2_prep module for a MLEV-4 pattern. However, our initial exams in six volunteers found similar image quality using either 3 or 4 180° pulses. Further optimization of the sequence is necessary to minimize B_0 _and B_1 _inhomogeneity while maximizing the sensitivity to oxygenation changes in the coronary system in more subjects. This will involve using different number of 180° pulses (even and odd numbers) with the same vasodilation condition in the same subject. Animal study will likely be ideal for such optimization purposes.

Rather than using a blood pool contrast agent (not approved in human study) as in our previously reported animal study [16], the FDA-approved extracellular agent Multihance was used in this human study to quantifying MBF and MBV, using a fast quantification method, a Consolidated-basis deconvolution. Comparing with the commonly used Fermi deconvolution, the Consolidated-basis deconvolution is more versatile and less sensitive to noise. This is because the Fermi method forces the impulse response to have an idealized shape - for example it is always monotonically decreasing, and positive. The Consolidated-basis does not have this guarantee. Multihance was selected in our human study because of its relatively high T_1 _relaxivity of 9.7 mM^-1^sec^-1^. The increased relaxivity derives from weak and transient interactions of the Multihance chelate with serum albumin. It appears that such transient interactions may confer a partial blood pool effect which serves to reduce the rate of contrast extravasations compared to other gadolinium based extracellular agents [25]. In the current human study, both resting and hyperemic MBF agrees well with other literature values.

### Limitations of this study

In this initial study, the primary goal was to investigate whether the T_2_prep method could yield similar hyperemic global myocardial OEF and MVO_2 _as the TSE method. Regional variations of these parameters were not assessed because neither OEF nor MVO_2 _map was created. However, such spatial variability needs to be measured in order for clinical evaluation of ischemic hearts. Precision and accuracy of this new cardiac oxygenation method using the T_2_prep technique have yet to be determined. A large sample of subjects with repeated measurements may be needed to estimate the precision, in combination with the noise propagation analysis. However, accuracy of the measurement has to be determined in animal studies to compare with the measurement results with a gold standard, such as blood oxygen content through arterial and venous blood sampling. A systemic hematocrit of 40% was assumed in this study and capillary hematocrit was also assumed to be 50% of coronary sinus. Because the parameters in Table 1 depend on the hematocrit, a small error in the calculation of myocardial OEF during the hyperemia may be introduced. If the capillary hematocrit varies by 25%, however, the calculated OEF variation is less than 3%. Another limitation of this method is the lack of regional myocardial oxygenation at rest. This is partially due to the fact that this method relies on endogenous deoxyhemoglobin, and its perturbation effects on the field inhomogeneity against the baseline condition (the so called BOLD effect). Other methods using exogenous agents, such as ^17^O-labelled gas [26] or contrast agents, may have the potential to resolve this problem. Lastly, CS measurement was not performed during the adenosine session due to limited vasodilation effect time (~4 min). Future study without using TSE sequence can allow estimation of CS oxygenation during the vasodilation.

## Conclusion

A modified CMR approach to estimate global myocardial oxygenation, in terms of myocardial OEF and MVO_2_, was designed and examined in human volunteers. The method has potential to assess regional myocardial OEF and MVO_2 _during hyperemia as well. The calculated global OEF and MVO_2 _appear to be consistent with other reported data. This method may be potentially useful for the assessment and management of patients with regional myocardial ischemia, as well as patients with altered global myocardial oxygenation, such as diabetes and obesity.

## Abbreviations

CMR: cardiovascular magnetic resonance, FLASH: fast low-angle shot, MBF: myocardial blood flow, MBV: myocardial blood volume, MVO_2_: myocardial oxygen consumption rate, NSF: nephrogenic systemic fibrosis, OEF: oxygen extraction fraction, ROI: region of interest, RPP: rate-pressure product, SR: saturation recovery, SNR: signal-to-noise ratio, T_2_prep: T_2 _preparation, TrueFISP: fast imaging with steady-state precession

## Competing interests

The authors declare that they have no competing interests.

## Authors' contributions

KSM, RJG, JZ participated in the design of the study. RO designed and implemented CMR sequence. ML, DL, and PKW enrolled, coordinated, and participated in the human study. KSM and JZ performed the CMR study and statistical analysis, as well as drafted the manuscript. All authors read and approved the final manuscript.
